# Acute Psychosis in Withdrawal from Nicotine Vaping in a Young Man with Comorbid Diabetic Ketoacidosis and Cannabis Use

**DOI:** 10.1155/2020/5710810

**Published:** 2020-06-03

**Authors:** Kyle J. Rutledge, Dianne L. Plath

**Affiliations:** ^1^Central Michigan University College of Medicine, Department of Psychiatry, 1000 Houghton Ave, Saginaw MI 48602, USA; ^2^Aleda E. Lutz Veterans Affairs Medical Center, Mental Health Annex, 4241 Barnard Road, Saginaw MI 48603, USA

## Abstract

Electronic delivery of nicotine, also termed “vaping,” has a growing evidence base suggesting potential harm through both exposure and withdrawal effects. The current report presents the case of a young man with multiple medical comorbidities, including insulin-dependent diabetes mellitus on an insulin pump and presumed Gilbert's disease, and chronic cannabis use who experienced acute agitation with hallucinations in the context of quitting his vape pen “cold turkey” or abruptly discontinuing use without a nicotine replacement. While undergoing hospitalization for his complaint of nausea and vomiting in the context of euglycemic diabetic ketoacidosis and cannabis use, his psychotic symptoms became evident and continued until beginning a nicotine replacement patch. A few months later, the patient returned to the hospital again for nausea and vomiting in the context of euglycemic diabetic ketoacidosis and reported cannabis use, however this time without psychosis, and notably after resuming and continuing use of e-cigarette with nicotine replacement delivered on admission. This is the first reported case of acute psychosis related to abrupt vaping withdrawal and adds to the plethora of information regarding potential risks associated with electronic cigarette use.

## 1. Introduction

Tobacco and nicotine products have a contentious history, with mainstream use and development of new delivery systems consistently outpacing medical evidence of the negative, disease-manifesting consequences of their use. Smoking tobacco products has been linked to psychosis, though typically the conversation revolves around increased incidence of smoking in patients with schizophrenia, as well as more difficulty with smoking cessation [1]. Myriad information has been documented on the association between daily tobacco use and risk of psychosis including during adolescence [2–4]. In contrast, rather little evidence has been systematically presented with respect to smoking cessation and psychosis, limited to positive case reports [5] and a small follow-up study yielding null results [6]. The use of more concentrated nicotine from modern electronic cigarettes (e.g., e-cigarettes and vape pens), also known as “vaping,” has been tied to a new wave of lung disease with associated deaths, according to the Centers for Disease Control and Prevention [7]. With emerging literature on the harmful elements of vaping usage and withdrawal [8], to date, no cases of psychosis related to withdrawal from vaping have been documented. The current case report presents a young man admitted to the medical hospital for euglycemic diabetic ketoacidosis with multiple medical comorbidities, cannabis use, and no psychiatric history, who experienced a cluster of symptoms including acute psychosis in the context of abrupt discontinuation of vaping nicotine or “quitting cold turkey.” A better understanding of this possible relationship—the potential harm of abruptly stopping concentrated nicotine with no replacement therapy—may enhance evaluation and counseling of patients across settings, including the emergency room, inpatient medical wards, outpatient clinics, and psychiatric hospitals.

## 2. Case Presentation

JD is a 19-year-old Caucasian male with pertinent history of insulin-dependent diabetes mellitus on an insulin pump and unconfirmed Gilbert's syndrome with no psychiatric history who presented to the emergency department with a chief complaint of nicotine withdrawal, stating “I quit vaping cold turkey yesterday.” The patient stated that he had been vaping—using electronic nicotine cigarettes—with increasing frequency over 6-9 months and using it “all day,” unable to quantify further, until abruptly discontinuing. Over the course of one day of acute withdrawal, he gradually developed nausea with vomiting, as well as significant anxiety, agitation, and irritability. He denied recent illness, travel, fevers, chills, abdominal pain, or diarrhea. JD revealed that as his somatic symptoms of withdrawal were intensifying, he also began to hear voices, reporting them starting at home and occurring again the night of his presentation. Medical history included one prior uneventful hospitalization for diabetic ketoacidosis (DKA) within a few years. The patient also had a history of probable Gilbert's disease, without genetic testing confirmation, diagnosed by an outpatient gastroenterologist during a visit with thorough examination and testing—including physical examination, abdominal ultrasound, C-reactive protein, and additional labs to rule out hemolysis—that were within normal limits with the exception of showing a consistent trend of elevated bilirubin. Further substance use included marijuana vaping; however, he discontinued this one week prior to presentation with no emergence of symptoms consistent with that timeline or any previous periods of sobriety he disclosed, and he denied any illicit drug use. JD's only prescribed medication at home was his insulin aspart pump. At the time of evaluation, JD had finished his first year of college including playing on the football team.

On examination, the patient was agitated with diaphoresis but otherwise well-appearing of athletic build, well-groomed, and with appropriate hygiene. Vital assessments were stable, and he was consistently afebrile, normotensive, and with adequate oxygen saturation. Physical exam was unremarkable for head/eyes/ears/nose/throat, neck, and cardiovascular, respiratory, gastrointestinal, musculoskeletal, and neurological systems. Lab results showed anion gap metabolic acidosis (venous pH 7.30, pCO_2_ 48, PO_2_ 51, HCO_3_ 23, anion gap 20, and CO_2_ 23) with ketones in urine and blood and beta-hydroxybutyrate elevated at 3.73 but normal blood glucose (122) and lactate (1.3). JD also displayed leukocytosis with white blood cells over 15,000, as well as hyperbilirubinemia with initial bilirubin 4.9 and indirect bilirubin also elevated (3.6). Eight-panel urine drug screen was positive for THC only, with serum alcohol < 0.01. Otherwise, blood tests including lactate and TSH, as well as amylase, lipase, electrolytes, liver enzymes, C-reactive protein, calcium, magnesium, and phosphate, were unremarkable. Blood cultures were drawn and negative x2 days; otherwise, no infectious disease panels were performed. Imaging included CT of the abdomen and pelvis which was negative for any acute process such as abdominal infection. No imaging of the head or other specialized neurological testing such as EEG was performed. JD was initially treated for euglycemic DKA with 1 L bolus of normal saline, followed by D5 normal saline with insulin drip and admission to the intensive care unit.

The psychiatry team was consulted to evaluate JD's agitated behaviors and psychotic symptoms. He reported that the voices began several hours after discontinuing vaping and continued into the evening in the hospital despite ongoing otherwise effective treatment for his ketoacidosis. Although formal assessment (e.g., MoCA and MMSE) was not performed at the time, JD remained alert and oriented, appearing at his cognitive baseline during the course of these symptoms, as confirmed by his mother present at bedside and overnight nursing staff. For nicotine withdrawal, he had initially declined nicotine replacement but on his second day requested a patch. Notably, his experience of voices began to subside after agreeing to initiate a nicotine patch that morning. The hallucinations resolved within a day. He denied any recent mood symptoms beyond his irritability following discontinuation of vaping and denied any history of hallucinations outside of the recent events, even during his previous hospitalization for DKA. Throughout the 3-day course of admission and treatment for his metabolic acidosis following the standard DKA protocol, his overall condition improved, with repeat labs showing a trend toward normalization and clearance by the endocrinology team for discharge home. His insulin pump was examined and did not require adjustment. He was not started on any psychotropic medications, though he was provided with information to pursue outpatient therapy for nicotine/substance use and school-related stressors.

Four months later, JD returned to the hospital with worsening nausea and vomiting over the course of one day in the absence of recent travel or other symptoms beyond some nasal congestion. At the time, he revealed that he had resumed use of e-cigarettes after his last hospitalization, with the most recent use just prior to presentation. He was again treated for euglycemic DKA after being found to have elevated urine ketones, serum beta-hydroxybutyrate (1.31), and anion gap 14 with normal glucose (108) and lactate (1.0). Bilirubin was also elevated at 4.2. Abdominal series showed no acute process, and abdominal ultrasound demonstrated thick bile sludge without stones but was otherwise normal. The remaining labs were unremarkable as well. A drug screen was not performed at that time, though he did report continued marijuana use. The treatment course followed the same trajectory as his prior hospitalization, with the exception that a nicotine replacement patch was applied earlier on in this episode, at the time of admission. With this presentation being otherwise the same as his last with the notable difference being that he did not experience nicotine withdrawal, remarkably, he also did not exhibit or describe any associated irritability or hallucinations during this hospitalization.

## 3. Discussion

To our knowledge, this is the first case reported of psychosis emerging in abrupt withdrawal of nicotine by vaping. The exact mechanism through which JD's psychosis emerged is unclear, as he experienced several possible contributing factors including euglycemic DKA, THC-positive urine drug screen, and hyperbilirubinemia, each of which has in their own right been tied to psychosis [9, 10]. However, the argument that nicotine withdrawal was an independent contributor to his psychosis is strengthened as the clinical presentation was both preceded and followed by other episodes of ketoacidosis with hyperbilirubinemia requiring hospitalization outside the context of acute e-cigarette nicotine withdrawal, where the patient did not exhibit agitation or psychotic features. Recent reports have started to uncover inflammatory, harmful effects of additives within e-cigarette vapors [7], which may have also contributed to the patient's expression of psychotic features beyond a purely nicotinic withdrawal etiology. Regarding his THC use, it is further possible that he had exposed himself to other illicit substances that did not appear on the drug screen which he either was unaware or chose not to disclose, but the fact that he was entirely psychiatric symptom-free for several days following his last use makes a substance-induced psychosis less likely. Also, the rapid resolution of hallucinations and agitation with the introduction of a nicotine replacement patch makes withdrawal from any other potential substances a less likely explanation for his symptoms, as these were not replaced. Finally, elevated bilirubin has been associated with both abrupt nicotine cessation [11] and psychosis [10]; however, with JD's history of Gilbert's syndrome and documented hyperbilirubinemia in the absence of any other psychotic events, elevated bilirubin is a less likely sole causal factor for his psychosis, though it may potentially act as a mediator.

As the case presented here is complicated by multiple factors, some additional limitations of his clinical picture must be acknowledged. First of all, it is possible that his ongoing cannabis use and reported abstinence for days prior to presentation may independently yield an effect on his level of attention, with confusion, and even the emergence of psychotic features. However, he reported a history of multiple other periods of sobriety that were not marked by somatic or psychotic features. It was not confirmed with drug screening whether he had recently used cannabis prior to his second hospitalization where he was not exhibiting agitation or psychotic features, though he reported daily marijuana use at the time. Second, other possible disorders on his differential diagnosis—including Wilson's disease or autoimmune diseases which may also cause agitation with psychosis—were not directly ruled out with testing, brain imaging, or specialized infection panels. However, with respect to Wilson's disease, suspicion remained low as he lacked family history, he demonstrated no hemolytic anemia, C-reactive protein was within normal limits suggesting no inflammatory or autoimmune process, and Kayser–Fleischer rings/jaundice or other physical manifestations were not evident. Third, whether the patient's clinical display is more consistent with delirium remains unanswered. Formal neurocognitive assessment was not performed, and as with alcohol withdrawal, it may be that his psychotic features were a manifestation of a broader delirium picture. As causes of delirium are often multifactorial and dynamic, it is also unclear how much his medical comorbidities including euglycemic DKA and recent cannabis use contributed to this delirium. As the presentation of delirium and psychotic symptom emergence in patients will vary based on multiple factors, including global clinical severity, comorbidities, medications, and intoxication, caution must be taken in interpreting the case presented here.

JD is clinically complex despite his age, and his chronic medical comorbidities likely impacted the emergence of his acute psychosis. In total, the lack of availability of additional objective measures as discussed above—including brain imaging and immunological investigation with his first hospitalization, or toxicology screening with his second visit reported here—leaves many possibilities from the differential diagnosis not entirely ruled out. Therefore, the main limitation of the case reported here is the difficulty in disentangling the specific contributions of each factor in the manifestation of JD's psychotic presentation. However, the tight temporal association between his initiation and resolution of acute psychosis corresponding with abrupt cessation and replacement of nicotine following an extended period of significant concentrated nicotine use through e-cigarettes suggests withdrawal of concentrated nicotine may have been a contributing factor for his psychosis which suggests a unique effect that has yet to be described in literature. This case report also contributes to the ever growing evidence for health risks associated with e-cigarettes as well as potential risks of quitting concentrated nicotine product use by the “cold turkey” method. As nicotine delivery becomes more concentrated, individuals have the capacity to subject themselves to much higher doses than we have seen previously; therefore, we must be prepared to encounter new clinical features of acute nicotine use and withdrawal. Future research can further investigate this relationship between concentrated nicotine withdrawal and psychiatric symptoms and continue to isolate harmful effects of all components of concentrated nicotine products available in vape pens.

## Data Availability

Data supporting the conclusions within this report are maintained in a confidential, secure electronic medical record, which, for purposes of patient privacy, cannot be released and will remain inaccessible to curious readers.
